# One factor to bind them all: visual foraging organization to predict patch leaving behavior with ROC curves

**DOI:** 10.1186/s41235-025-00624-7

**Published:** 2025-04-05

**Authors:** Marcos Bella-Fernández, Manuel Suero Suñé, Alicia Ferrer-Mendieta, Beatriz Gil-Gómez de Liaño

**Affiliations:** 1 Universidad Autónoma de Madrid, Madrid, Spain; 2 UNIE Universidad, Madrid, Spain; 3 Universidad Pontificia de Comillas, Madrid, Spain

**Keywords:** Visual foraging, Optimal foraging theory, Organization, ROC curves, Composite variables

## Abstract

Predicting quitting rules is critical in visual search: Did I search enough for a cancer nodule in a breast X-ray or a threat in a baggage airport scanner? This study examines the predictive power of search organization indexes like best-r, mean ITD, PAO, or intersection rates as optimal criteria to leave a search in foraging (looking for several targets among distractors). In a sample of 29 adults, we compared static and dynamic foraging. Also, we reanalyze data from diverse foraging tasks in the lifespan already published to replicate results. Using ROC curves, all results consistently show that organization measures outperform classic intake rates commonly used in animal models to predict optimal human quitting behavior. Importantly, a combination of organization and traditional intake rates within a unitary factor is the best predictor. Our findings open a new research line for studying optimal decisions in visual search tasks based on search organization.

**Supplementary Information:**

The online version contains supplementary material available at 10.1186/s41235-025-00624-7.

## Significance statement

Predicting quitting rules in search is critical to most visual search tasks. Did I search enough for a cancer nodule in a breast X-ray or a threat in a baggage scanner at the airport? Did I look hard enough for my friend in the crowd? Making optimal decisions on when to leave a search is a crucial problem in psychological science. In this study, we examined the predictive power of search organization as an optimal criterion to leave a search, comparing it with classic intake rate measures commonly used in foraging. Using ROC curves, our results show that organization measures outperform intake rates to predict optimal quitting behavior, but importantly, a combination of organization and classic intake rates within a unitary factor seems the best predictor of leaving decisions. Our findings open a new research line for studying optimal decisions in visual search tasks based on search organization knowledge.

**Supplementary Information:**

The online version contains supplementary material available at 10.1186/s41235-025-00624-7.

## Introduction

Imagine being a coin or stamp collector attending a fair or a flea market. You find a promising stand and start searching for pieces to add to your collection. After some time searching, you decide to leave that stand and go to the next one. This is a daily instance of a visual foraging task: search for targets (some given coins or stamps) in a display (a pool of diverse coins or stamps) for an undetermined time, and leaving after deciding that searching there is no longer worth it.

Visual foraging tasks like these are increasingly being studied in psychological science, partly because they are more naturalistic than classical visual search tasks. Among other interesting characteristics, an essential aspect of search tasks, is the spatial organization or systematicity needed to find a target. Spatial organization consists on exploring a given zone or display through a particular strategy, which ideally avoids unnecessary eye movements and refixations of already explored locations (Klein & McInnes, 1999; Li et al., 2023; but see Hooge et al., 2005). Visual search has a systematic component in the sense that there is a non-random bias in search scan paths (Gilchrist & Harvey, 2006). As in other aspects of daily life, organization is important in foraging because more organized searches tend to be more efficient (Bella-Fernández et al., 2024; Ólafsdóttir et al., 2021; Smith & De Lillo, 2022). From a cognitive perspective, studying organization in visual foraging may add useful insights regarding cognitive processes like planning future behavior or visual memory strategies.

Typically, organization in visual foraging (or cancelation tasks) is measured through four indicators: best-r, the mean inter-target distance (ITD), the percentage above optimal scan path (PAO), and the number of intersections between non-consecutive scan paths (Bella-Fernández et al., 2024; Ólafsdóttir et al., 2021; Woods et al., 2013). These indicators capture different aspects of spatial organization, but the empirical evidence suggests that all these measures share a significant amount of variance, being intimately related to describe organization patterns in search (Woods & Mark, 2007; Woods et al., 2013). Although other indexes have been described recently, such as those using Bayesian sampling (Clarke et al., 2022), these four are the most widely used because of their strong empirical support (Guilbert, 2024; Ólafsdóttir et al., 2021; Woods et al., 2013; see Bella-Fernández et al., 2024, for a comparative use these organization indicators in foraging).

Another critical aspect of visual foraging in non-exhaustive tasks is the quitting criterion. That is, when and why the observer decides that search has come to stop in a given setting to continue searching in a new one. Quitting criterion has been studied in the context of visual search and other scenarios where items may be absent (e. g. Chun & Wolfe, 1996), as well as situations where may targets are available (e. g. Kristjánsson et al., 2020; Wolfe, 2013). In this last context, the importance of studying leaving criteria is twofold: the quitting decision influences the task itself and is a relevant instance of decision making. With respect to the first one, leaving too early may cause the observer to miss important targets and leaving too late would make the observer to spend more time than necessary. Thus, Harhen and Bornstein (2023) found that observers tend to “overharvest” in foraging tasks and related this trend with the informative effect of foraging more time than optimal.

Several quitting rules have been proposed in the context of animal foraging, like the fixed giving-up time rule or other assessment rules based on the quality of patches and iterative guesses of optimal thresholds (Bella-Fernández et al., 2022; Lloyd et al., 2023). However, the Marginal Value Theorem (MVT; Charnov, 1976) is the most widely used on animal and human foraging. MVT is based on the optimal foraging theory (OFT) and poses that observers tend to forage optimally in terms of resource intake. MVT predicts that observers would leave the current search when the instantaneous intake rate of the last item picked/selected drops below the average intake rate of that given search (Charnov, 1976; Wolfe, 2013). Importantly, in free-leaving foraging tasks the observers do not tend to exhaustively deplete the displays if they can leave them earlier, even in the cases when they are encouraged to collect all the targets (e. g. Wolfe, 2013, experiment 2). Although some data suggest that human visual foragers tend to slightly deviate from optimality (Gil-Gómez de Liaño et al., 2022; Harhen & Bornstein, 2023; Kristjánsson et al., 2020), it seems to be a quite satisfactory theory to understand quitting rules in foraging so far (Wolfe, 2013).

The behavior of the organization in foraging shares two main characteristics with the intake rates. The intake rate is linked to the efficiency in the sense that, as a trial advances, targets become scarcer and search efficiency decreases; similarly, organization is related to efficiency in the sense that organized search tend to be more efficient (Ólafsdóttir et al., 2021; Smith & De Lillo, 2022). Furthermore, like resource intake, we have recently showed that organization also tends to decrease across a foraging trial (Bella-Fernández et al., 2024). Of course, a possible explanation is that organization decreases simply because there are less available targets. However, our recent evidence suggests that organization is usually lower when displays start with more targets (Bella-Fernández et al., 2024). Thus, less targets do not necessarily mean lower organization. Then, the way the organization indicators change throughout a foraging search in a given environment before moving on to a new one (or just stopping the search) seem to be good candidates to operate as rule-predictors of foraging quitting decisions.

However, as seen above, there is not a unitary organization index, but different indicators with varying measurement scales. Due to their varying rationales, choosing only one and discarding the rest might affect the validity of the organization measure. From a theoretical perspective, it would be interesting to test how they behave independently as quitting rules predictors, and eventually, combine them into a composite individual index that could consider all of them with their peculiarities describing general search organization.

Our goal in the present study is twofold: First, we will study to what extent different search organization indicators can serve as potential meaningful predictors of quitting rules in foraging. To do so, we will apply receiver operating characteristic (ROC) curves to explore the existence of a threshold based on the organization measures for participants to make the decision of staying in the current display or leaving it. Second, we will test the validity of a single, composite organization measure combining all those classic measures together. We will compare the classic individual search organization indicators, and the composite single one. Importantly, we will also test classic OFT intake rate indicators typically used to predict quitting rules in foraging with our composite and organization indicators. To do so, we will use the data of three visual foraging studies. Study 1 used a sample of 29 young adults (18–28 years old) who performed three foraging tasks: static foraging, dynamic foraging with a moderate speed, and dynamic foraging with a fast speed, using original empirical unpublished data. Study 2 uses a sample of 97 participants 5–38-year-old performing dynamic foraging under different memory load conditions. These are re-analyzed data from Gil-Gómez de Liaño and Wolfe (2022). Last, Study 3 tests classic feature-conjunction conditions in a sample of 279 observers from 4 to 25 years old. These are also re-analyzed data from Gil-Gómez de Liaño et al. (2022) and Bella-Fernández et al. (2024). Following, we will show empirical evidence that some organization indicators and our single composite index can be even better predictors of quitting rules in foraging than classic OFT intake rate indicators, as predicted by ROC curves.

## Methods

### Study 1

The goal of this study is to find evidence of the usefulness of organization measures on predicting the decision of staying or leaving in the current patch to start a new search. We tested the predictive power of search organization in leaving decisions between static and dynamic foraging tasks, as it can be a critical factor to consider in foraging tasks organization.

#### Sample

The sample of this study consists of 30 young adults, university students from 18 to 28 years old in Madrid. Participants provide data from several trials per condition, resulting in sample sizes of hundreds or thousands of data per ROC curve, sufficient for a proper parameter estimation (Hanley & McNeil, 1982). Nonetheless, the sample size was not pre-calculated since we did not know the effect size for the current ROC analyses and thus cannot calculate power. All participants had normal or corrected-to-normal vision. Each participant gave informed consent before their participation. The participants completed the Conner’s Continuous Performance Test, version 3 (CPT-3; Conners, 2014). In this 15-min test, letters appear in the center of the screen and the participants had to respond by pressing the spacebar as quickly as possible once the letter appeared. They had to respond to every letter except X, where they had to omit any type of response, in a go-no-go task in which the exposure times of the stimuli vary throughout the task. We used this task to corroborate the absence of significant attention disorders in the participants.

Only data from participants without signs of attention disorders according to the CPT-3 were selected to these analyses. Participants were excluded if they obtained high scores (*t* ≥ 60) in one of the following variables from the CPT-3: omissions, commissions, discriminability, hit reaction times (hit reaction times with *t* ≤ 45 were also exclusion criteria), hit reaction time standard deviation, and variability. One participant was discarded after the assessment with the CPT-3. Thus, the final sample comprised 29 university students from 18 to 28 years old (mean age = 20.23, sd = 2.10).

#### Equipment and stimuli

**Fig. 1:**
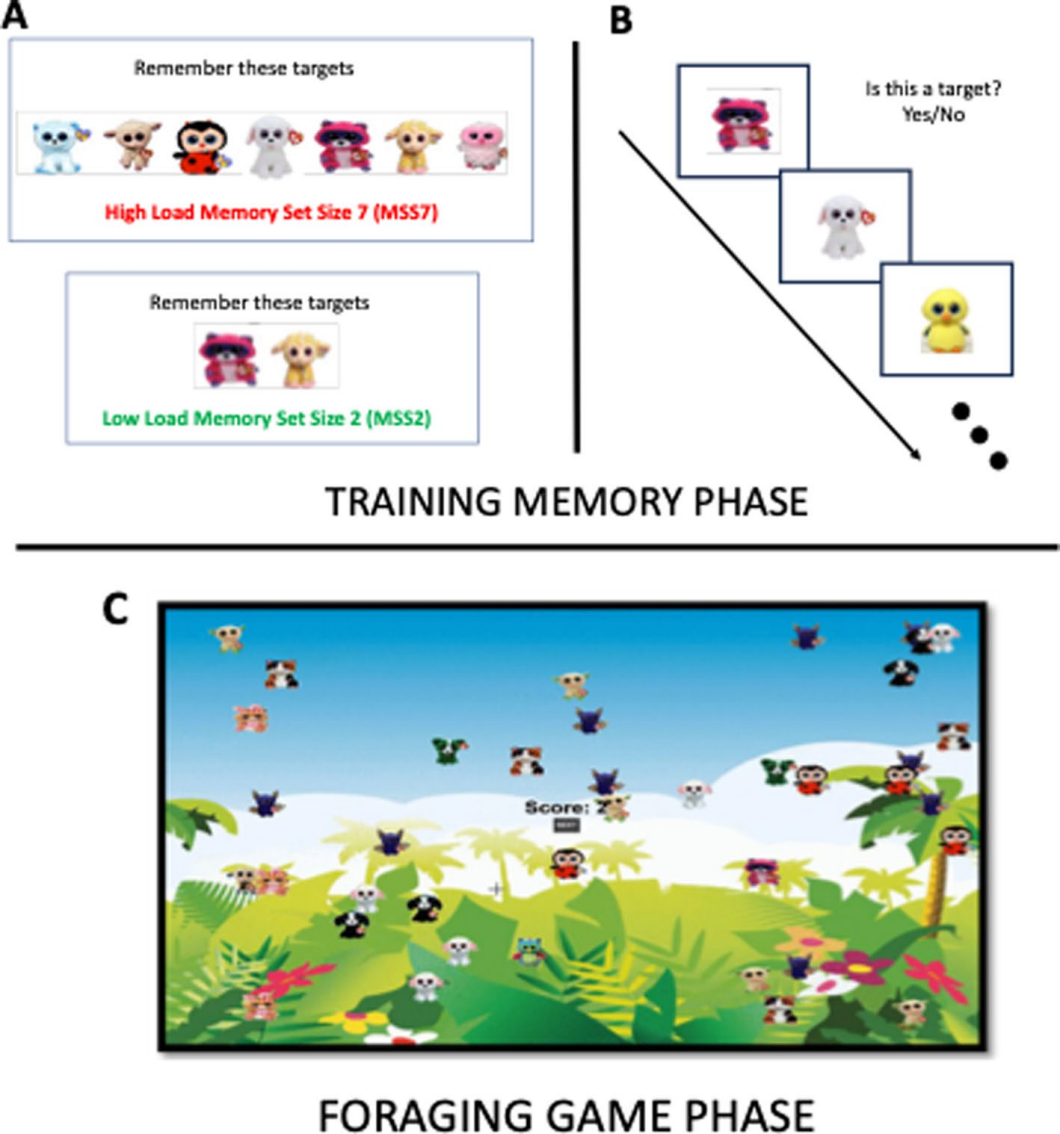
Instance of the three phases on the experimental procedure in studies 1 and 2: **A** First, the observer is shown the 2 or 7 targets, depending on the experimental condition, which they have to keep in memory and search for; **B** After that, the observer performs a recognition task, where they must recognize the targets with a minimum hit rate of 80% (otherwise, the observer is redirected to the phase **A**; **C** The observer has to search for the targets in the display, being free to leave the current display to face a new one at all times. Each targets gives 2 points, and errors take 1 point. The task lasts until the observer reaches 200 points. Reproduced with permission from Gil-Gómez de Liaño and Wolfe (2022)

In this study we used a Microsoft Surface pro i5 device with a 12.3-inch screen and a resolution of 1400 × 1500 pixels to perform the tasks. The main tasks were based on the serious video game^1^ developed by Gil-Gómez de Liaño and Wolfe (2022), in which a key variable was modified for the present study: dynamicity. The game uses the images of well-known children stuffed animals as stimuli, randomly selected from an image bank composed of 297 images. The toys were shown framed on an invisible rectangle of size 1.33° × 1.00°. They also appeared on a basic background of a jungle (Fig. 1) just like in Gil-Gómez de Liaño and Wolfe (2022).

The set size for each display could be 40, 80 or 120 stimuli, resulting in three conditions of set size. The memory load of the task was set at 5 target types that the participants had to search for under all possible conditions. Previous to the experiment itself, the participants had to memorize the target types and a brief recognition test was performed to be sure that the participants memorized them. In each display between 3 and 5 types of the set targets appeared. This represented 20–30% of the stimuli presented per patch. All of these were displayed on each screen and for each participant, although the targets to be searched for were always the same (5) within each participant, they varied between conditions (static & the two dynamic conditions) and participants.

The speed at which the stimuli moved was constant in each task, being 44 pixels/sec in the *Dynamic Slow* condition, 88 pixels/sec in the *Dynamic Fast* condition, and null (0 pixels/sec) in the *Static* condition. In both Dynamic conditions the stimuli changed direction at semi-randomized intervals.

#### Procedure

The experiment was conducted in a single session with an average duration of 60 min, depending on the speed of the participants in performing the tests, as well as the breaks taken between them. The task was divided into three parts, with each part reflecting a condition (static, slow and fast dynamic ones).

The instructions were presented to the participants on the screen, and explained by the experimenter. Next to them were displayed the 5 stuffed animals that would be the targets for each particular condition. First, they were presented all at once and then one by one. The participants then performed a recognition test on the targets in which they had to obtain at least 80% correct to be able to pass it and begin the search task; if they did not, they had the opportunity to memorize the targets and perform the memory test again until reaching that 80% correct.

Throughout the tasks, the targets were mixed with the distractors and the participants had to select them in order to score points, for each selected target they would get 2 points. After selecting them, the targets disappeared from the screen and, therefore, participants had to go through several screens in order to score the 200 points. If a distractor was selected, a red cross would temporarily appear over the distractor and it would subtract a point from the score.

The participants were free to leave the current display and start a new one at any time by tapping on the “next” button in the center of the screen. Any time the observers left a display they had to wait for a constant delay (or “travel cost”) of 2 s. Leaving a display had no cost in points. The decision of when to switch screens was made at the discretion of each participant, while maintaining the objective of getting the points as fast as possible. They were reminded in the instructions that it was not necessary to collect all the present targets to be able to switch patches. Before starting the experiment, the participants underwent a practice phase where they had to score 50 points using the same test targets.

The order of the conditions was controlled by randomizing the six possible orders of the combinations of the three conditions (static, slow, and fast). They had to reach 200 points in each condition, so the experiment would end as soon as the participant reached 600 points in total. In addition, and with the objective to avoid mixing targets among tasks, between each of the 3 conditions participants performed the CPT-3 test, mentioned above, and filled out a questionnaire about sociodemographic data (age, gender…). They were also given the opportunity to take a break to avoid major fatigue between tasks.

### Study 2

This study aims to replicate study 1 dynamic foraging, focusing on the performance of a large sample of children, adolescents, and adults already published: We re-analyzed the Gil-Gómez de Liaño and Wolfe (2022) data set but for the current purposes of the study; the organization measures and their predictability in decision rules in foraging. Following the study methods of that previously published research, we also assessed the effect of participants' memory load on the organization by comparing two memory load conditions (as we will follow in the methods) and their predicting capability of quitting rules.

#### Sample, equipment, and stimuli

Although all details about the methods can be found in Gil-Gómez de Liaño and Wolfe (2022), we will give details for the reader to understand the basic manipulations and essential methods to understand the experiment. They used a sample of 97 participants divided into three age groups: 5–6 years (34 observers), 11–12 years (30 observers), and + 18 years (33 observers ranging from 18 to 38 years old, Mean = 25.42). In all subgroups, half of the participants were male and half female. 5–6-year-old participants were tested with the Conners Kiddie Performance Test (K-CPT 2) and the rest of them were tested with the CPT-3, in order to discard attention deficits.

The apparatus and procedure were the same as those of study 1, except for the set sizes. The set sizes were 40, 80, and 120 now, and targets were 20%−30% of the items in each trial, regardless of the set size. Unlike in the former study, in this case the items were always moving in pseudo-random directions at a speed of 44 px/s (there was no static condition). In this study, two conditions of memory load are performed: an easy memory load condition in which observers should look for 2 targets, and a hard memory load condition with 7 potential targets to look for (in study 1, the memory load was 5 targets in all cases). These conditions were counterbalanced across participants. As the 7-target condition was considerably more difficult, and in order to control that the participants remembered the targets to look for, a memory task was implemented before the foraging task. Observers should remember that targets and they were tested in a recognition test before starting the foraging task. They must get 80% correct in the memory recognition test to advance to the foraging task. For more specific details, see Gil-Gómez de Liaño and Wolfe (2022).

#### Procedure

Every observer must obtain 200 points per memory load condition picking up targets by tapping on them using a touchable Surface i5 computer. Each target was rewarded with 2 points and every distractor cost 1 point. The score was visible on the screen. They were free to leave a display/patch to start another one at every time (by clicking on the “next” button on the center of the screen, see Fig. 1). There was also a constant time gap of 2 s of travel-time between displays. Leaving a display had no cost in points. Conditions were run separately pseudo-randomly assigned and counterbalanced for the observers, with a total of 400 points (200 for each condition), plus a 50-point practice session for each condition (thus, a total of 100 points of practice). The 5–6-year-old participants were asked for the instruction to make sure that they understood the task before the end of the practice block. The whole session, including the CPT-3 or K-CPT, the practice block, and the two experimental conditions, lasted for about 30 to 45 min.

### Study 3

The purpose of this study is to obtain further evidence of the findings of previous studies 1 and 2, but using different types of stimuli in dynamic foraging. So, we re-analyzed the Gil-Gómez de Liaño et al. (2022) data set (see also Bella-Fernández et al., 2024) in which 279 children, adolescents, and young adults performed dynamic foraging tasks using squares and circles of different colors. Thus, we compared the predictive capability of organization measures in a different type of foraging tasks of those used in studies 1 and 2, particularly in classic feature and conjunction foraging tasks.

#### Sample, equipment and stimuli

Although all details about the methods can be found in Gil-Gómez de Liaño et al. (2022), we will give details for the reader to understand the basic manipulations and essential methods to understand the experiment. Thus, the sample and data come from that experiment, guaranteeing a final sizeable cohort at each range of age of between 21 and 33 observers at each age group from 4 to 25. The sample is composed of 279 observers from 4 to 25 years old (mean = 10.14, sd = 5.09) from elementary, middle, high school, and college in Madrid. All participants had normal or corrected-to-normal vision. Parents or guardians gave written informed consent for each participant, and all gave also verbal or written consent upon their age. None of the participants had neurological or sensorial damage, motor impairments, or a diagnosis of schizophrenia or generalized developmental disorder.

Before entering the experiment, the participants were assessed with several attention and executive function tests: CPT-3 or K-CPT 2, the Behavioral assessment scale for children (Reynolds & Kamphaus, 2004), the Behavior Rating Inventory of Executive Function (Gioia et al., 2000), and the Reynolds Intellectual Screening Test (Reynolds & Kamphaus, 2003). Participants with attention impairments or an intellectual quotient blow 70 were discarded for the experiment (see Gil-Gómez de Liaño et al., 2022 for details).

The code was programmed in MatLab 7.10 using the Psychophysics Toolbox, version 3 (Brainard, 1997; Kleiner et al., 2007; Pelli, 1997). Targets and distractors were squares for the feature condition (green, blue, yellow, and red), and squares and circles for the conjunction condition (green and blue). Every display contained 60, 100, 140, or 180 items, with a proportion of targets randomly generated between 20 and 30% for each trial. Items appeared in random locations across the display, starting locations and movement directions randomly changed from trial to trial. The items were moving in random directions at 44 pixels/sec to make systematic searches more difficult. Instead of speed or memory load, the conditions in this experiment were Feature foraging or Conjunction foraging: in the feature condition, both targets and distractors has the same shape (squares) and only differed in color, while in the Conjunction condition targets and distractors differed in both shape (circles or squares) and color.

#### Procedure

**Fig. 2:**
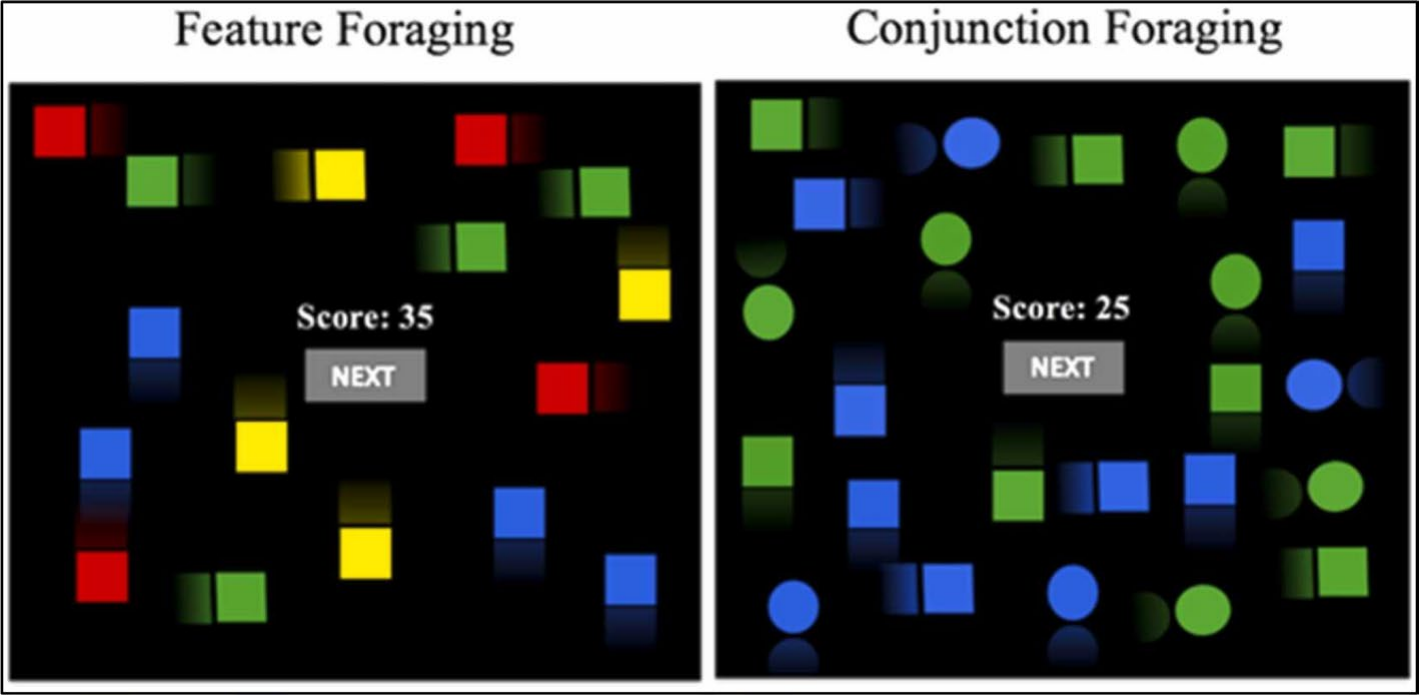
Examples of Feature and Conjunction displays similar to those from study 3. In Feature foraging, only one feature (color) differences targets and distractors. In Conjunction foraging, both color and shape have to be considered when discriminating between targets and distractors. Reproduced with permission from Gil-Gómez de Liaño et al. (2022)

Participants had to collect targets as fast as possible until obtaining 200 points per condition (a total of 400 points for each feature and conjunction conditions), plus a previous 50-point practice block each. Every target correctly collected gave 2 points, and every distractor collected subtracted 1 point. When a correct target was tapped, it disappeared from the display. Distractors did not disappear when collected, but a red cross appeared to represent a commission error. In the feature condition, targets were blue and green squares and distractors were red and yellow squares. In the Conjunction condition, targets were green circles and blue squares, and distractors were green squares and blue circles. Items were smaller than those used in studies 1 and 2. On each trial, set size was random with possible set sizes of 60, 100, 140, and 180 items, with a random 20% to 30% of targets. The observers were free to leave the search at any time by clicking a “next” button in the center of the display; then, after waiting for a 2-s delay (a “travel cost”), a new display appeared for the observer. Leaving a display has no cost in points. The whole task lasted for about 20–40 min. Figure 2 shows the feature and conjunction foraging tasks for study 3.

### Statistical analysis

#### Organization measures and RIAIR

The organization indicators used in our work are those used mainly in the visual search field: best-r, the mean ITD, the PAO, and the intersection rate between scan path segments.

The *best-r* takes its name from “*the best correlation*” with “up-down” and “left–right” search patterns within a search. In a given display, once the targets are collected, we can assign the value “1” for the first collected target, “2” for the second one, “3” for the third one, and so forth, generating an “order of collection” variable. The screen display coordinates *x* and *y* of each target can be associated with those targets, making it possible to calculate the correlation between the order of target collection and the coordinates *x* and *y*. The best-r is the largest (in absolute value) of these two correlations. The best-r captures the trend of the observer to persevere in either horizontal or vertical scan directions (that is, “up-down” and “left–right” search patterns, as we mentioned above).

**Fig. 3:**
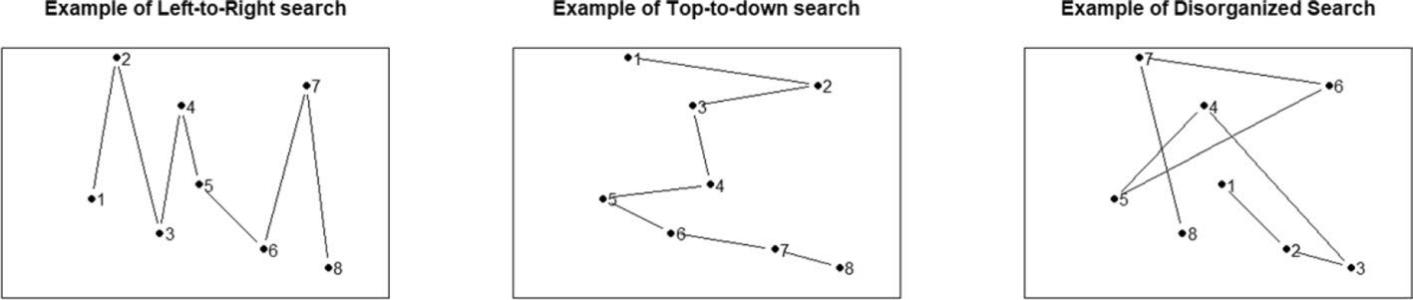
Examples of left-to-right, top-to-down, and random search, respectively. The two first spatial strategies are scan-like strategies, and their associated best-r are close to 1. The third example shows a disorganized search with a moderate best-r value

Figure 3 graphically shows the best-r calculation. The three panels have the targets (dots) in the same locations, but the collection order is different. The leftmost panel shows a reading-like strategy, collecting targets from the leftmost to the rightmost. The best-r of this example is 0.991. The second panel shows a top-to-down collecting strategy, whose related best-r is 0.980. The rightmost panel shows a random strategy, and its associated best-r is 0.393.

The *mean ITD* is a simple measure calculated by computing the arithmetic mean of the distances between consecutively collected targets.

The *Percentage Above the Optimal Path (PAO)* is a more complicated measure, especially in motion searches. To calculate the PAO, we need to obtain the optimal path between the locations of the targets collected; mathematically, this is a form of the *Traveling Salesman Problem* (McGregor & Ormerod, 1996). The actual path length is divided by the optimal path length. Then, we must subtract one from that ratio to calculate how often the actual path length is higher than the optimal one and multiply it by 100 to obtain an index of a percentage. Equation 1 describes the PAO calculation.

1 $${\text{PAO}} = \left( {\frac{{{\text{Actual}}\;{\text{path}}\;{\text{length}}}}{{{\text{Optimal}}\;{\text{path}}\;{\text{length}}}} - 1} \right)*100$$

**Fig. 4:**
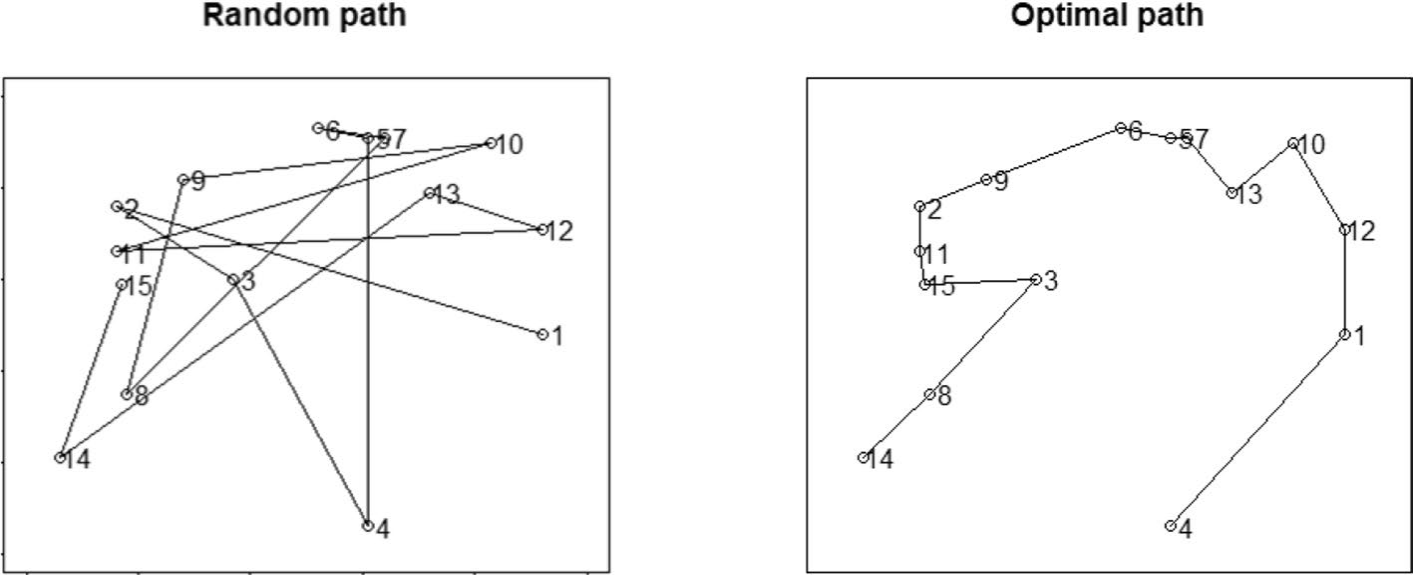
Random and shortest paths from the same target locations. Even without calculations, it is observable that the random path and the inter-target distances are considerably larger than the optimal path and its inter-target distances. The Mean ITD and the PAO are sensitive to inter-target lengths. Taken from Bella-Fernández et al. (2024)

Although in motion foraging it is more difficult to obtain PAO, making a new optimal path calculation after picking a given target (that usually takes a few hundreds of milliseconds), one for every target picked within a patch, has shown similar reliable values of PAO measures (Bella-Fernández et al., 2024). Both the mean ITD and the PAO are measures based on the path length and the idea that the shorter the path length, the better the organization. Figure 4 shows examples of a random search and the shortest path (optimal path) of the same target locations.

Lastly, *the intersection rate* is the number of intersections between scan path segments divided by the number of segments. The intersection rate is based on the idea that organized searches do not return to previously inspected areas. Figures 3 and 4 show that paths from random searches have more intersections than paths from organized searches. More detailed descriptions of the indicators are available elsewhere (Bella-Fernández et al., 2024; Ólafsdóttir et al., 2021; Woods et al., 2013).

On each trial (also referred as patch or display, with a given number of targets and distractors in every set size condition), an observer can decide to leave the current display to move on and search in a new potentially more profitable one, after getting desirable targets. This decision of staying/leaving will be the predicted variable. Note that this dependent variable is dichotomic (staying/leaving). The predictors are the organization indicators and the intake rates. Also note that every single response is used as a decision: response immediately preceding a leaving behavior are considered as “leaving” decisions”, while the rest of the responses (with subsequent target collected before leaving) are considered “staying” decisions.

For each target within a trial, from the third one picked, we can calculate organization indexes associated to the “trial so far”.^2^ Therefore, we can study the decision made for every target collected up to a particular one. For instance, if an observer has taken 10 targets in a trial, we can calculate the organization variables considering the 10 targets. But we can also consider only the first 9 targets and associate the values to that 9th target, or the first 8 targets, or the first *n* targets, being *n* ≥ 3). This way, we can observe the intra-trial progress of the organization indicators in a more detailed way, looking for variabilities that might predict quitting rules.

Finally, we calculated the intake average rate and instantaneous rate of every target picked within a trial following MVT. The MVT predicts that a forager would stay in the patch while the instantaneous intake rate is greater than the average intake rate, and that the forager would leave the current patch as soon as the instantaneous intake rate drops below the average intake ratio. In other words, the forager would stay in the patch as long as the Ratio Instantaneous intake rate / Average Intake Rate (RIAIR) is larger than 1, and they would leave the display as soon as the RIAIR drops below 1. Remember that the main objective of the study is to determine the best way to predict the decision whether an observer decides to move on to the next trial, based essentially on organization measures, but also comparing them with classic foraging theories like MVT widely used to understand quitting rules. Thus, we also calculated those intake rates based on the MVT to compare how MVT’s intake rates and organization indicators work predicting the decision of staying/leaving during foraging.

**Fig. 5:**
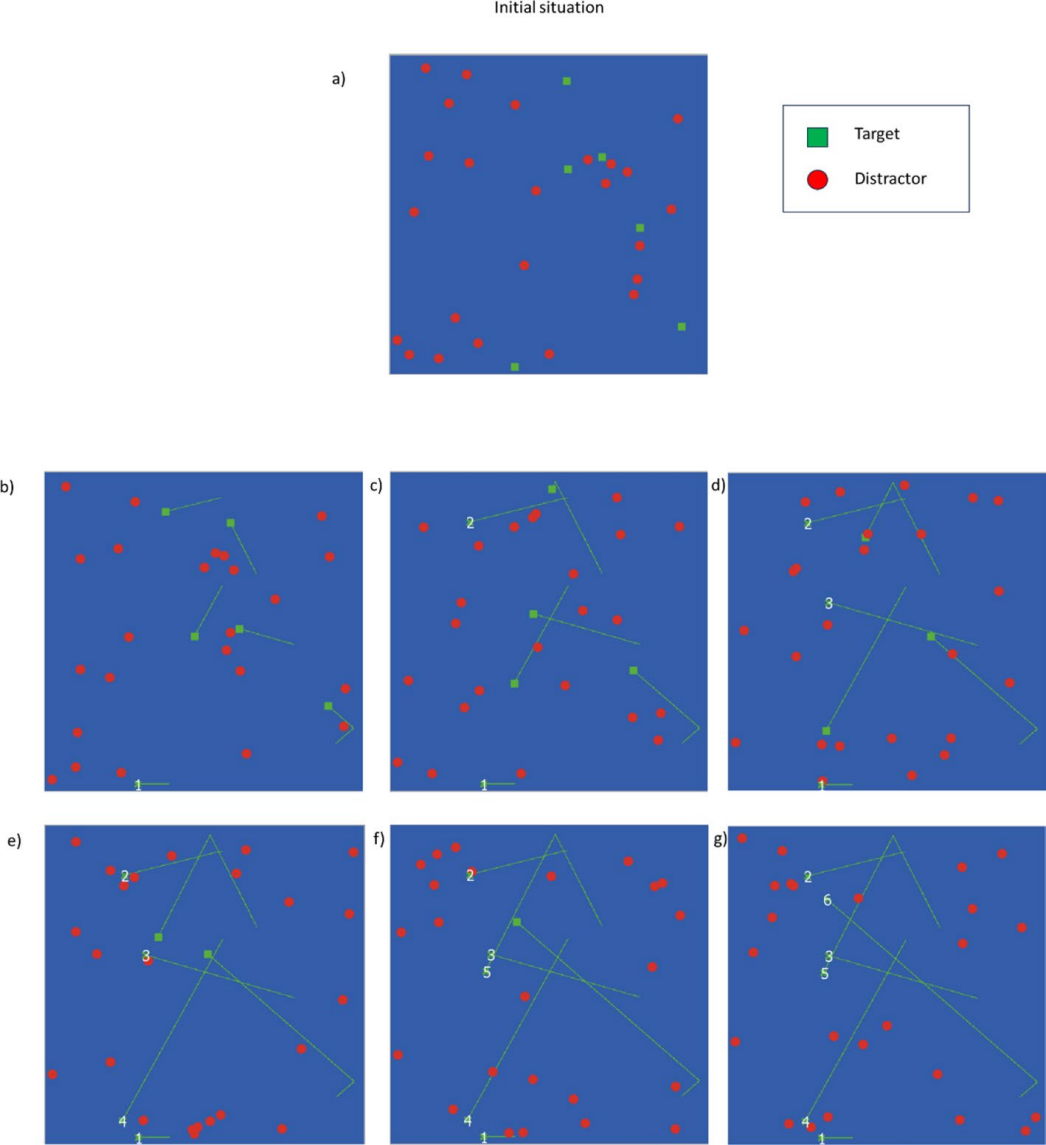
A made-up example of a dynamic foraging trial. **a** The display starts with targets and distractors placed in random locations. **b** The items moved in pseudo-random direction at a constant speed until a first target (labeled 1) is captured. **c** The uncollected targets keep moving and a second target (labeled 2) is also collected. **d**–**g** The targets are subsequently being captured. **g** All the targets have been collected and the coordinates of the locations where they were captured had been recorded. These coordinates are the ones used to calculate the organization variables

Finally, it is important to consider that calculating these organization indicators needs the coordinates of the target positions. Obtaining these coordinates from static foraging task is straightforward, but it is not for dynamic tasks. In these cases, to calculate the organization indicators we took the coordinates of each target at the instant it was collected. Figure 5 illustrates the process with a made-up, simplified example. Panel a) shows a plausible initial situation with 6 targets (green squares) and 22 distractors (red circles). In this case, the observer is following a left-to-right strategy. Panel b shows the position of the first collected target. The location of the first target at the capturing moment is recorded. Note that all the targets had moved from their initial position (green straight lines). The first target has been collected, so it disappears; in this figure, we keep its location represented. Panel c shows the capture of the second target; again, the reader could note that the targets had moved, except the previously captured one. Panels d to g show the capture of the rest of the targets, with their trajectories drawn until the instant of their respective collections. In Panel g all the targets have been collected, and the locations where they had been captured had also been recorded. These location coordinates were those used to calculate the organization measures, as they have been successfully applied in previous research studying organization measures in dynamic foraging (see Bella-Fernández et al., 2024).

### Principal component analysis (PCA) and composite variables

We were interested in determining whether a one-dimensional solution is legitimate to group the four organization indicators. Then, we performed PCA to further assess convergent validity for the four organization indicators; visual foraging organization is, indeed, conceptualized as a unitary construct (Ólafsdóttir et al., 2021; Woods & Mark, 2007). But an exploratory factor analysis reported by Woods et al. (2013) showed that best-r and Mean ITD could load on different, although correlated, factors, potentially measuring different aspects of search organization compared to the other indicators. To perform the PCA, we considered the organization measure values calculated at the end of each trial, because using more than one value per trial would violate the assumption of independence between observations. We also contemplated the possibility of performing a confirmatory factor analysis, but the assumptions of normality and the moderate multicollinearity were violated, leading to unreliable results and Heywood cases.

One of the main objectives of the present work was to determine whether a composite variable joining all four organization indicators here tested could be a good unitary measure for search organization in our foraging tasks. Song et al. (2013) describe several ways to obtain a composite variable. We calculated the weighted averaging method including the loadings of each variable on the first component from the PCA as weights. According to Song et al. (2013), we need first to standardize the variables to be composited.

In our case, for each organization measure we took all the instances calculated from all “trials so far”: The organization measures were calculated after *n* targets, that is, all the values calculated for every target picked on each trial, starting from the third target of each trial. This way, we obtained four new variables for each organization index (best-r, mean ITD, PAO, and intersections), which are now the *standardized* best-r, *standardized* mean ITD, *standardized* PAO, and *standardized* intersection rate. Then, the weighted composite organization variable, named as C_w_, is simply the sum of the standardize variables for each participant multiplied by each index-load derived from the PCA analysis, following Song et al. (2013), and as described in Eq. 2. We used the loadings on the first component obtained from the PCA as the weighting coefficients (w in Eq. 2) for every measure to correct their contribution for the organization unitary index. The Mean ITD, PAO, and intersection rate indicators have negative signs since their interpretation as organization indicators is based on a negative relationship (the lower the index the higher the organization), while best-r follows a direct positive interpretation (the higher the best-r the higher the organization); Thus:

2 $$C_{w} = w_{{{\text{best}} - r}} *z_{{{\text{best}} - r}} - w_{{{\text{mean}}\;{\text{ITD}}}} *z_{{{\text{mean}}\;{\text{ITD}}}} - w_{{{\text{PAO}}}} *z_{{{\text{PAO}}}} - w_{{{\text{intersection}}\;{\text{rate}}}} *z_{{{\text{intersection}}\;{\text{rate}}}}$$

### Receiver operating characteristic (ROC) curves

**Fig. 6:**
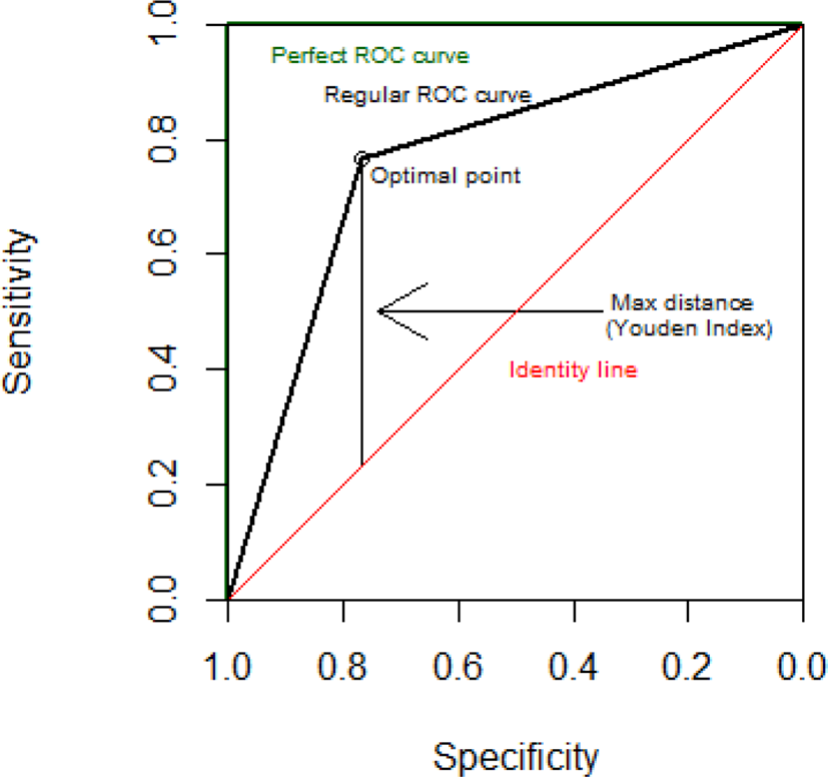
Example of ROC curve and its associated optimal threshold

ROC curves are a widespread method to assess the potential of a certain factor to discriminate between the two categories of a dependent variable, like staying/leaving dichotomic decisions as those used in our study. To draw a ROC curve, we use the values of the factor as a tentative decision threshold where the predictions are testable against the actual decisions. Then, sensitivities (true-positive rates) and specificities (true-negative rates) can be obtained from each threshold; of course, more conservative thresholds would tend to bring lower false-positive rates at the cost of also bringing lower true-positive rates. The ROC curve is the curve which results from taking the sensitivities and specificities pairs, drawing them into a two-axis graph: the vertical axis (by convention) being the sensitivity and the horizontal axis being the specificity. Both sensitivity and specificity range from 0 (null sensitivity or specificity) to 1 (perfect sensitivity or specificity). Figure 6 provides a graphical explanation of ROC curves. A perfect predictor variable has a ROC curve overlapping with the y-axis and a horizontal line at the top of the curve domain (green line in Fig. 6). In that case, the area under the curve (AUC) is 1, which would be the index to determine a perfect prediction. At the other end, a useless predictor, equivalent to guessing or making random decisions, has a curve close to the Identity line (red line in Fig. 6) and has an area under the curve close to 0.5. AUC values of 0.70 are considered moderate-to-good levels of predictability (Hosmer et al., 2013). Usually, ROC curves (black line in Fig. 6) fall between the perfect ROC curve (green line) and the Identity line (red line). Besides the ROC curve indexes to test how good the prediction is, we can also calculate the optimal threshold that gives us an idea of the predictive results. That optimal threshold is the farthest point of the ROC curve from the identity line, and the distance is called the Youden index (Perkins & Schisterman, 2006).

Threshold models have been useful in optimal stopping models in general and search tasks in particular (Baumann et al., 2020; Sang et al., 2020). Indeed, the classic foraging theories like the MVT are mostly threshold models; remember that MVT states that the optimal foraging behavior threshold comes when the instantaneous intake rate of the last item picked drops below the average intake rate across a given search (trial in our case). That threshold would be the best moment to move on and leave the search according to MVT, that can also be calculated by ROC curves.

Under this framework of deciding when it is the optimal moment to leave a search, sensitivity denotes the ability to predict the decision of leaving the search, while specificity evaluates the ability to predict the decision of staying in the patch. We performed ROC curves using each organization indicator, composite variable or RIAIR as a predictor, and the dichotomous decision variable “stay” or “leave” as the predicted variable. For instance, if best-r can take values from 0 to 1, every value between 0 and 1 can be a predictive threshold which separates decision between “stay” and “leave” decisions. Then, sensitivity and specificity can be obtained by contrasting these predictions with the actual decisions; the sensitivity is the proportion of predicted “leave” decisions which are actually correct, and the specificity is the proportion of predicted “stay” decisions which are actually correct. To obtain a large number of points for the ROC curves, many thresholds of the organization indicators are tested and their associated sensitivities and specificities are obtained; these points then compose a single ROC curve for each indicator, condition, and study. Then, we obtained the point with the largest Youden index (see Fig. 6) in each ROC curve as the optimal threshold.

Both the PCA and the ROC curve estimations were performed separately for each condition on each study: in Study 1, we calculated the three ROC curves associated with each speed condition (static, low, and fast); in Study 2, we split the data in two age groups (children on one group, adolescents and young adult on the other group) and also in the two memory load conditions (2 and 7 target types), with a total of four ROC curves; in Study 3, we again split the data in the same two age groups than in Study 2, and also for Feature and Conjunction conditions, with a total of four ROC curves. Note that for age, the threshold of 10 years is not arbitrary: between 9–10 years old, many attentional processes related to visual search and visual foraging experience important changes, clearly differentiating both groups (Gil-Gómez de Liaño & Wolfe, 2022; Gil-Gómez de Liaño et al., 2020). Indeed, youngest children tend to be more disorganized (Bella-Fernández et al., 2024; Guilbert, 2024; Ólafsdóttir et al., 2021), and those of 4–5 years old tend to leave searches a bit earlier than children over 6 years and adults; with adults (and, even more, older adults) usually overharvesting (Gil-Gómez de Liaño & Wolfe, 2022; Gil-Gómez de Liaño et al., 2022; Wiegand et al., 2019). Also, previous work (Kristjánsson et al., 2020) shows that observers tend to leave easier feature searches with higher collection rates compared to the more difficult conjunction ones. Although it would also be interesting, set size was randomly selected for each participant at each patch in the experiments, so we cannot consider it as a controlled variable, and unfortunately we do not have enough set size trials (patches) for all participants to study it as another factor.

### Combining ROC curves

The purpose of combining the ROC curves into a single one is to explore the possibility of combine the predictive properties of the RIAIR and the organization composite variable. The results (see below) show that RIAIR tends to be a good predictor of staying decisions, while the organization tends to predict well the leaving decision. Combining the two of them could result in a good predictor of both decisions.

We combined the two ROC curves associated with the weighted composite variable and the RIAIR. We combined them into a single ROC curve, following the method proposed by Haker et al. (2005). This method starts from the sensitivities and specificities of each predictor at every point of their respective ROC curves and, through computing their associated maximum likelihood estimations; that method provides combined sensitivities and specificities of both indexes, and, with them, generates a combined ROC curve.

**Fig. 7:**
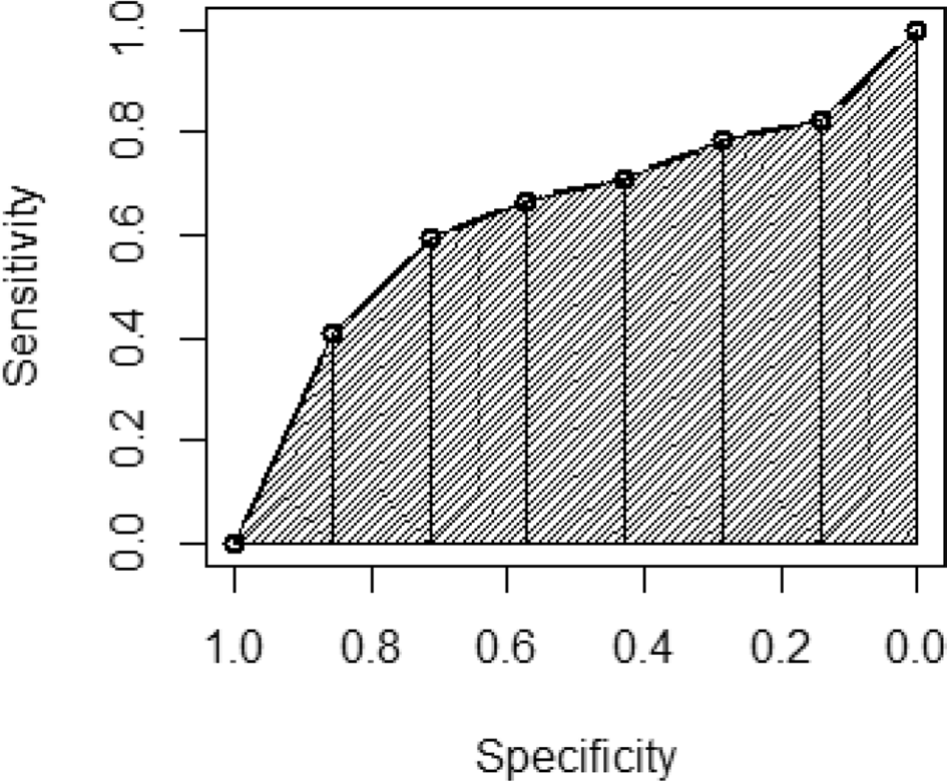
Example of the Trapezoid rule for estimating AUCs. The horizontal axis is divided in a sufficient number of intervals. Then, for each interval, we can draw trapezoids whose right bases are the horizontal axis intervals and whose oblique bases are the respective dots in the ROC curve. Afterward, we sum the trapezoid areas and take this sum as an approximation of the AUC

Because Haker et al. (2005) do not specify a direct method to estimate the AUC of the combined ROC curves, we used the trapezoid rule from sensitivities and specificities described by Fawcett (2006). Figure 7 shows a simulated example of how the trapezoid method is implemented. The ROC curves are drawn in two-dimensional spaces with the sensitivities in the vertical axis and the specificities in the horizontal axis. Each couple of sensitivity–specificity is a point in the two-dimensional space. The ROC curve is the curve crossing all these points (solid black line in Fig. 7). We then can draw a right trapezoid with two consecutive sensitivity–specificity points and their respective projections on the horizontal axis. Drawing these right trapezoids (dashed polygons in Fig. 7) between every pair of consecutive points and then calculating the area of all the trapezoids allow us to get a good approximation to the actual AUC.

The software used to estimate ROC curves from the data include methods to estimate the confidence intervals for AUCs. Alas, we cannot directly use the same software to combine ROC curves. Instead, we applied a method from Hanley and McNeil (1982) to estimate the confidence intervals for the AUC of the combined ROC curves (See Supplementary Material for a description of this method).

### Software

We used RStudio for R to develop all the analyses. We used the packages “paran” (Dinno, 2018) for parallel analysis, “ForagingOrg” (Bella-Fernández, 2022) to compute the organization measures, and “pROC” (Robin et al., 2011) to calculate the ROC curves.

## Results

### Parallel analysis, PCA, and composite organization variable calculations

Although our first objective was to study how general organization measures could be good predictors of quitting rules decision in search, we have also considered the possibility to include a single unitary organization measure combining the four indicators as described in the methods.

The parallel analysis in the three studies suggested a one-dimensional factorial structure.

Table 1 summarizes the results of the parallel analysis in the three studies and the PCA split by condition and age group. The parallel analysis and the PCA^3^ show that fitting all four indexes into a one-factor model is acceptable in the three studies. This evidence allows us to group the four indicators into a composite variable.

**Table 1:** Parallel analysis and PCA in the three studies

	Parallel analysis: adjusted Eigenvalue	PCA: percentage of variance explained by the first component (%)
Study 1		
Static	1.85	49.05
Dynamic slow	2.19	67.83
Dynamic fast	2.04	63.67
Study 2		
Memory set size 2, Children	2.30	73.79
Memory set size 2, Adolescents and Adults	2.18	66.97
Memory set size 7, Children	2.03	66.99
Memory set size 7, Adolescents and Adults	2.27	67.50
Study 3		
Feature, Children	2.51	72.84
Feature, Adolescents and Adults	2.62	77.91
Conjunction, Children	2.26	57.64
Conjunction, Adolescents and Adults	2.26	75.50

### ROC curves

#### General results

We compared the predictiveness of individual organization indicators and the weighted composite organization variable on the leaving decisions in foraging by using ROC curves, as explained in the statistical analyses. As we can see in the methods, all three studies are a rich source of different factors, since the samples include children from early childhood to early and mid-adulthood. Also, in study 1 we have new data from both static and dynamic tasks, while in study 2, the data come from two memory load conditions (2 versus 7 targets to look for in the foraging task; easier and harder, respectively, than the memory load of 5 used in study 1). In study 3, we can see data from two different classic foraging conditions: feature and conjunction foraging searches.

**Fig. 8:**
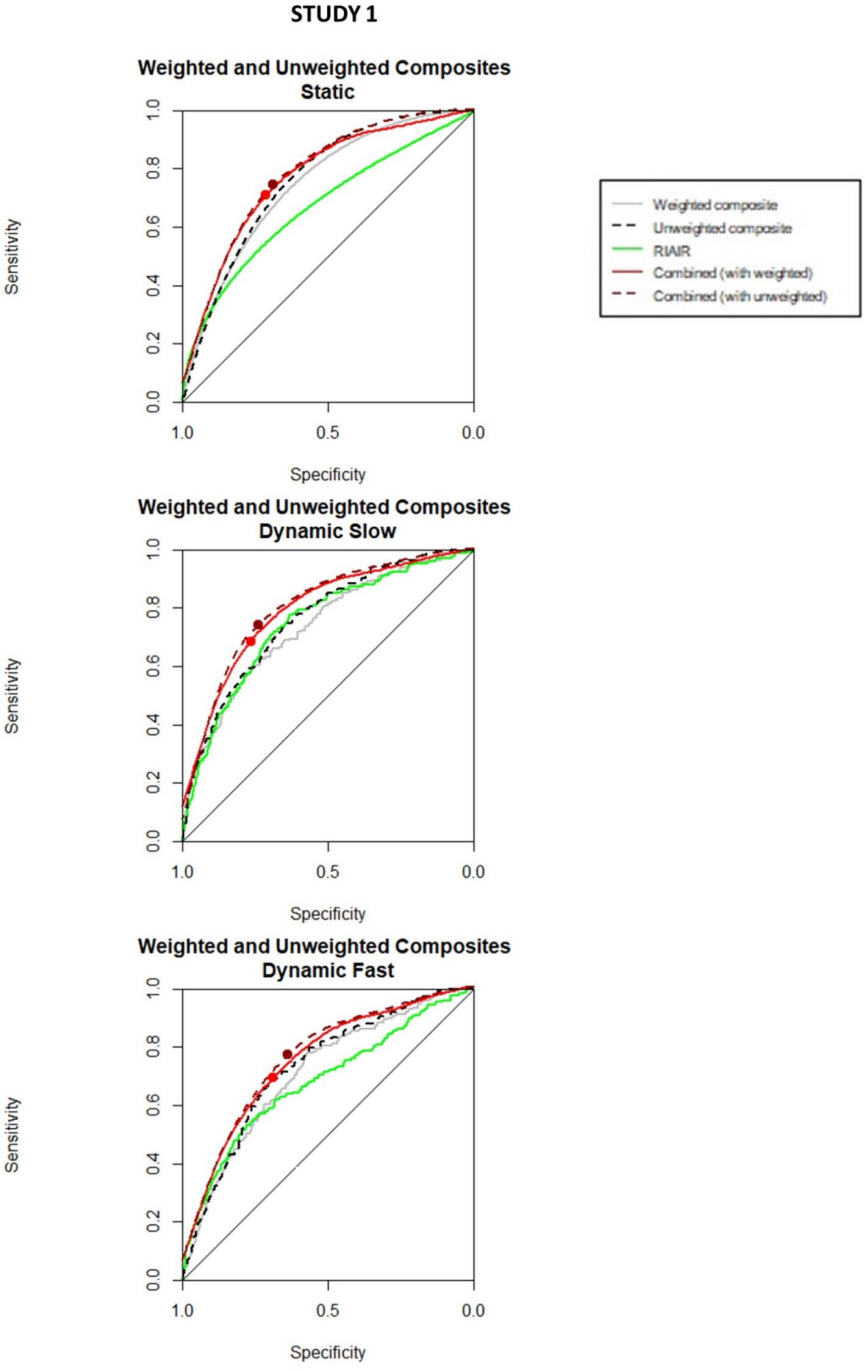
ROC curves for composite organization variables, RIAIR, and combined ROC curves in Study 1 split by condition (static, slow, and fast). The big dots in the combined ROC curves mark their respective associated optimal thresholds. The ROC curves were smoothed for graphical purposes, with no impact on calculations

**Fig. 9:**
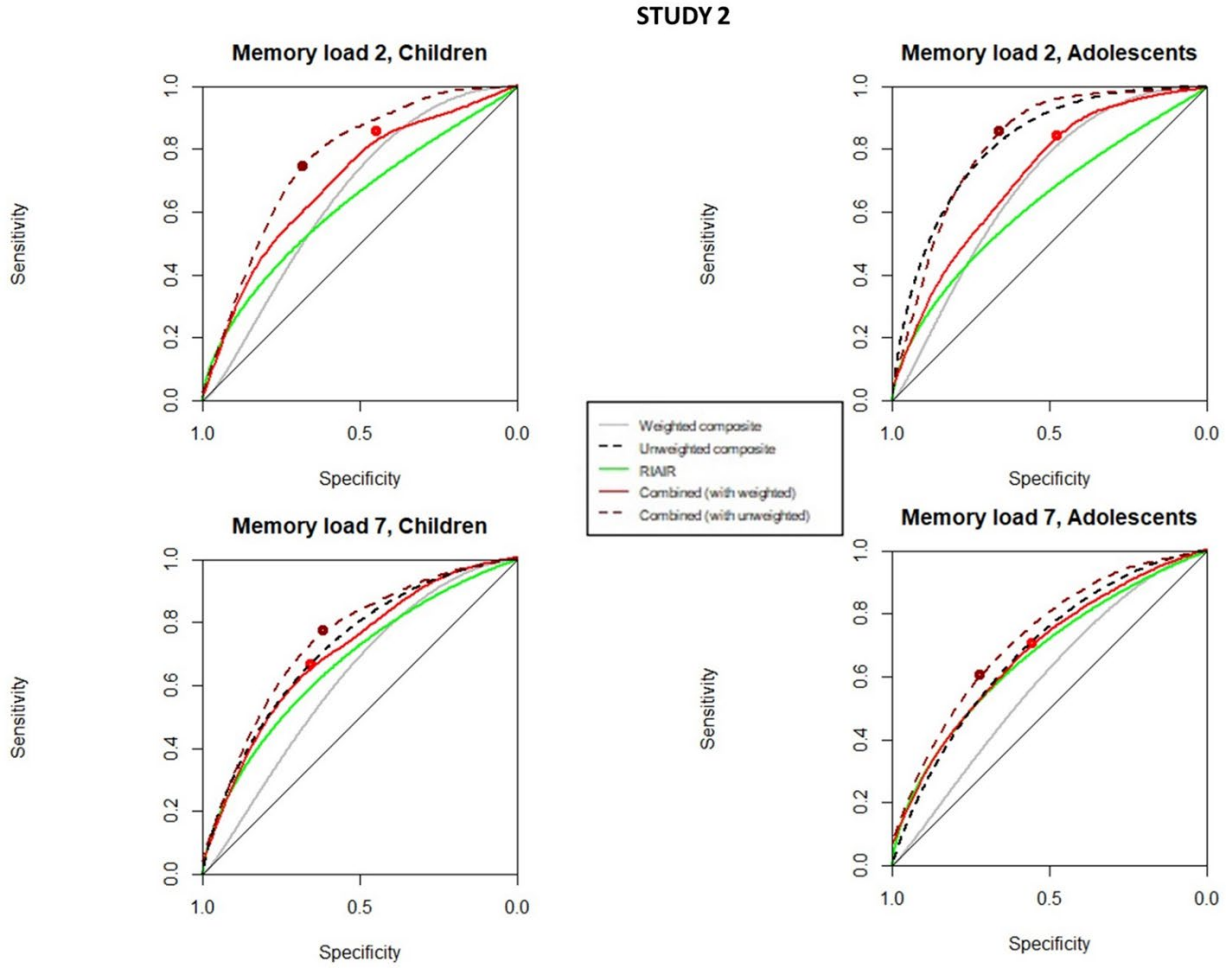
ROC curves for composite organization variables, RIAIR, and combined ROC curves in Study 2 split by memory load condition (2 vs. 7) and age group (children under 10 vs. adolescents and adults over 10). The big dots in the combined ROC curves mark their respective associated optimal thresholds. The ROC curves were smoothed for graphical purposes, with no impact on calculations, and thus some of the optimal thresholds appear slightly out of the smoothed curves

**Fig. 10:**
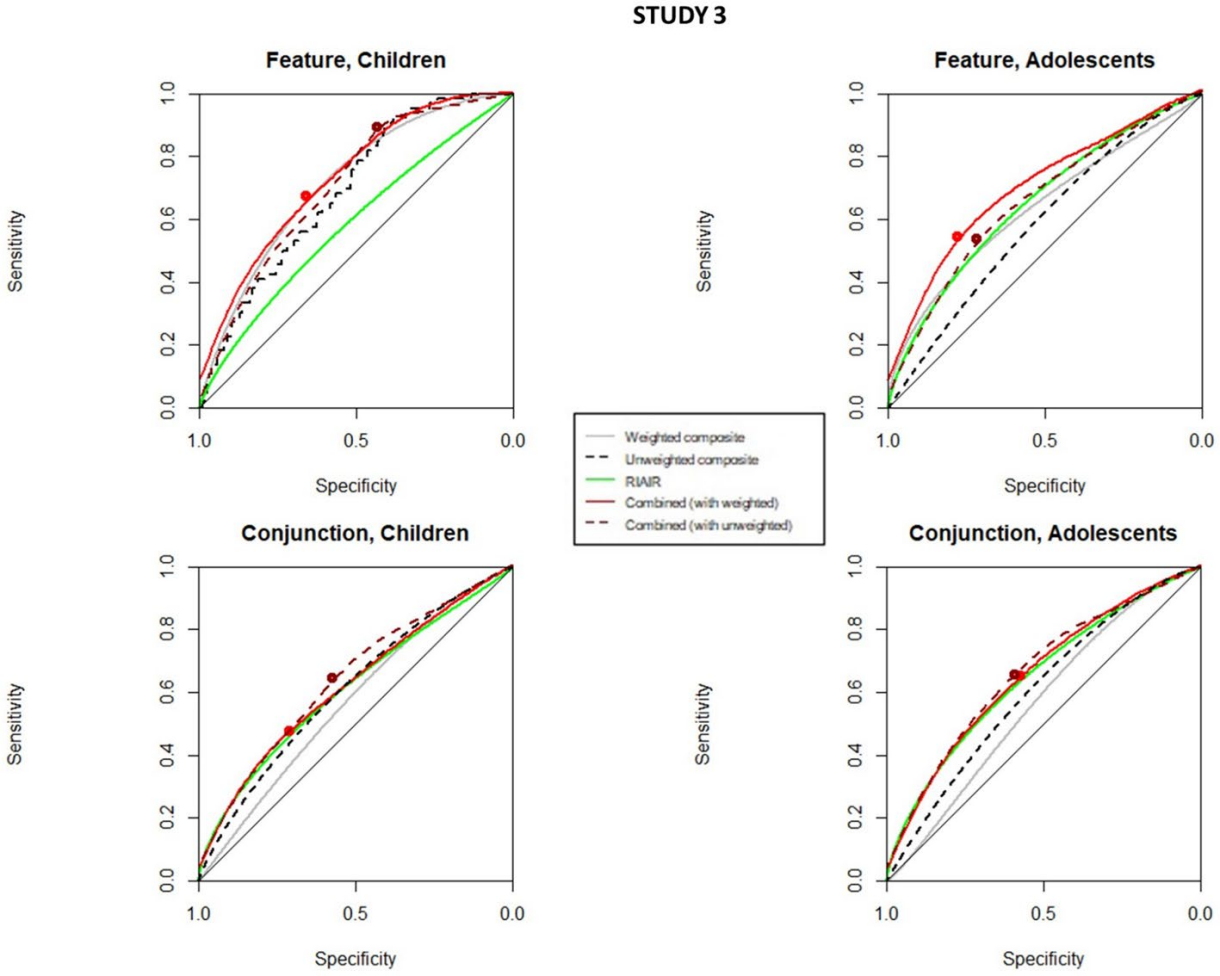
ROC curves for composite organization variables, RIAIR, and combined ROC curves in Study 3 split by condition (feature vs conjunction) and age group (children under 10 vs. adolescents and adults over 10). The big dots in the combined ROC curves mark their respective associated optimal thresholds. The ROC curves were smoothed for graphical purposes, with no impact on calculations, and thus, some of the optimal thresholds appear slightly out of the smoothed curves

Thus, we performed ROC curves analysis splitting the data by condition (static, slow, and fast for study 1, 2–7 memory load conditions in experiment 2, and feature and conjunction in study 3), and age group (< 10 years and > 10 in studies 2 and 3), as also detailed in the methods section. In Figs. 8, 9 and 10, we can see the ROC curves comparing age and conditions for studies 1, 2, and 3, respectively. Tables 2, 3 and 4 show the estimated parameters in those conditions, for all studies.

**Table 2:** ROC curves in study 1

	Static	Dynamic Slow	Dynamic fast
Best-r			
AUC (95% CI)	0.697 (0.655, 0.739)	0.703 (0.664, 0.742)	0.678 (0.642, 0.714)
Optimal threshold	0.735	0.561	0.598
Sensitivity	0.709	0.690	0.734
Specificity	0.645	0.656	0.550
Mean ITD (in pixels)			
AUC (95% CI)	0.695 (0.660, 0.730)	0.727 (0.693, 0.761)	0.688 (0.654, 0.721)
Optimal threshold	315	362	353
Sensitivity	0.814	0.799	0.869
Specificity	0.509	0.584	0.421
PAO			
AUC (95% CI)	0.717 (0.680, 0.754)	0.711 (0.670, 0.753)	0.679 (0.640, 0.718)
Optimal threshold	35.85	54.55	66.43
Sensitivity	0.703	0.632	0.598
Specificity	0.669	0.689	0.707
Intersection rate (in intersections/target)			
AUC (95% CI)	0.690 (0.649, 0.730)	0.694 (0.651, 0.737)	0.683 (0.645, 0.722)
Optimal threshold	0.087	0.372	0.298
Sensitivity	0.738	0.494	0.628
Specificity	0.562	0.803	0.677
Weighted composite			
AUC (95% CI)	0.751 (0.716, 0.785)	0.736 (0.697, 0.775)	0.712 (0.676, 0.748)
Optimal threshold	0.170	− 0.82	− 0.429
Sensitivity	0.634	0.626	0.774
Specificity	0.742	0.732	0.578
Unweighted composite			
AUC (95% CI)	0.760 (0.728, 0.793)	0.762 (0.727, 0.798)	0.731 (0.696, 0.766)
Optimal threshold	0.05	− 0.829	− 2.54
Sensitivity	0.628	0.741	0.658
Specificity	0.760	0.654	0.720
RIAIR			
AUC (95% CI)	0.676 (0.629, 0.722)	0.747 (0.709, 0.786)	0.682 (0.640, 0.724)
Optimal threshold	1.147	1.186	0.746
Sensitivity	0.663	0.776	0.533
Specificity	0.642	0.633	0.781
Combined Weighted composite and RIAIR			
AUC	0.773 (0.731, 0.815)	0.796 (0.756, 0.836)	0.757 (0.733, 0.781)
Optimal threshold	Composite < − 0.1 OR RIAIR < 0.5	Composite < 0.8 AND RIAIR < 1.2	Composite < − 2 OR RIAIR < 0.65
Sensitivity	0.712	0.687	0.698
Specificity	0.693	0.766	0.690
Combined Unweighted composite and RIAIR			
AUC	0.786 (0.763, 0.809)	0.807 (0.785, 0.829)	0.769 (0.745, 0.792)
Optimal threshold	Composite < − 0.3 OR RIAIR < 0.55	Composite < − 1.65 AND RIAIR < 1.3	Composite < − 2.55 OR RIAIR < 0.5
Sensitivity	0.749	0.743	0.775
Specificity	0.715	0.741	0.641

**Table 3:** ROC curves split for experimental condition (memory load) and age group for study 2

	Memory load: 2		Memory load: 7	
	Children	Adolescents and Young adults	Children	Adolescents and Young adults
Best-r				
AUC (95% CI)	0.687 (0.650, 0.723)	0.741 (0.712, 0.770)	0.667 (0.616, 0.718)	0.655 (0.625, 0.684)
Optimal threshold	0.392	0.411	0.245	0.449
Sensitivity	0.741	0.820	0.462	0.732
Specificity	0.589	0.572	0.806	0.501
Mean ITD (in pixels)				
AUC (95% CI)	0.662 (0.624, 0.700)	0.695 (0.664, 0.727)	0.623 (0.579, 0.668)	0.591 (0.560, 0.621)
Optimal threshold	397	343	408	357
Sensitivity	0.768	0.856	0.752	0.790
Specificity	0.554	0.493	0.457	0.368
PAO				
AUC (95% CI)	0.779 (0.743, 0.815)	0.821 (0.792, 0.849)	0.711 (0.664, 0.758)	0.675 (0.645, 0.705)
Optimal threshold	102	90	123	101
Sensitivity	0.804	0.796	0.598	0.559
Specificity	0.653	0.714	0.728	0.707
Intersection rate (in intersections/target)				
AUC (95% CI)	0.783 (0.747, 0.819)	0.821 (0.794, 0.848)	0.699 (0.650, 0.749)	0.673 (0.642, 0.705)
Optimal threshold	0.782	0.532	0.789	0.628
Sensitivity	0.703	0.814	0.650	0.546
Specificity	0.744	0.713	0.689	0.718
Unweighted composite				
AUC (95% CI)	0.774 (0.739, 0.810)	0.823 (0.797, 0.849)	0.725 (0.679, 0.771)	0.688 (0.659, 0.717)
Optimal threshold	− 2.024	0.014	− 2.455	− 0.051
Sensitivity	0.717	0.916	0.701	0.756
Specificity	0.709	0.604	0.673	0.526
Weighted composite				
AUC (95% CI)	0.653 (0.615, 0.691)	0.683 (0.651, 0.716)	0.616 (0.571, 0.662)	0.582 (0.551, 0.613)
Optimal threshold	− 0.038	0.441	− 0.017	0.178
Sensitivity	0.761	0.838	0.812	0.719
Specificity	0.542	0.484	0.390	0.425
RIAIR				
AUC (95% CI)	0.634 (0.581, 0.686)	0.626 (0.578, 0.674)	0.672 (0.619, 0.725)	0.669 (0.636, 0.702)
Optimal threshold	0.712	0.662	1.20	1.32
Sensitivity	0.442	0.389	0.675	0.651
Specificity	0.806	0.848	0.612	0.597
Combined Weighted composite and RIAIR				
AUC (95% CI)	0.664 (0.614, 0.714)	0.717 (0.673, 0.741)	0.648 (0.593, 0.703)	0.683 (0.648, 0.717)
Optimal threshold	Weighted composite < 0 OR RIAIR < 0.6	Weighted composite < 0.4 OR RIAIR < 0.4	Weighted composite < 0.8 AND RIAIR < 1.2	Weighted composite < 0.8 AND RIAIR < 1.7
Sensitivity	0.859	0.844	0.669	0.709
Specificity	0.450	0.479	0.661	0.557
Combined Unweighted composite and RIAIR				
AUC (95% CI)	0.741 (0.693, 0.789)	0.797 (0.756, 0.837)	0.735 (0.682, 0.787)	0.721 (0.688, 0.755)
Optimal threshold	Unweighted composite < − 2 OR RIAIR < 0.3	Unweighted composite < − 0.3 AND RIAIR < 5	Unweighted composite < − 2.4 OR RIAIR < 0.4	Unweighted composite < − 2.09 OR RIAIR < 0.6
Sensitivity	0.748	0.858	0.777	0.609
Specificity	0.685	0.661	0.619	0.722

**Table 4:** ROC curves split for condition and age group for Study 3

	Feature		Conjunction	
	Children	Adolescents and Young adults	Children	Adolescents and Young adults
Best-r				
AUC (95% CI)	0.596 (0.536, 0.656)	0.492 (0.439, 0.546)	0.558 (0.542, 0.574)	0.537 (0.516, 0.558)
Optimal threshold	0.757	0.808	0.319	0.469
Sensitivity	0.894	0.820	0.545	0.762
Specificity	0.335	0.303	0.544	0.307
Mean ITD (in pixels)				
AUC (95% CI)	0.714 (0.656, 0.772)	0.638 (0.568, 0.707)	0.568 (0.552, 0.584)	0.568 (0.549, 0.588)
Optimal threshold	278	302	303	275
Sensitivity	0.712	0.487	0.690	0.757
Specificity	0.622	0.765	0.417	0.364
PAO				
AUC (95% CI)	0.685 (0.629, 0.740)	0.573 (0.512, 0.635)	0.606 (0.590, 0.623)	0.630 (0.610, 0.650)
Optimal threshold	39	39	125	99
Sensitivity	0.818	0.590	0.549	0.702
Specificity	0.500	0.568	0.612	0.504
Intersection rate (intersections/target)				
AUC (95% CI)	0.638 (0.576, 0.700)	0.500 (0.440, 0.561)	0.608 (0.592, 0.625)	0.631 (0.611, 0.652)
Optimal threshold	0.186	0.098	0.975	0.676
Sensitivity	0.712	0.577	0.492	0.639
Specificity	0.544	0.453	0.676	0.580
Unweighted Composite				
AUC (95% CI)	0.690 (0.636, 0.745)	0.609 (0.592, 0.625)	0.588 (0.526, 0.650)	0.615 (0.595, 0.635)
Optimal threshold	2.96	− 2.35	2.71	− 0.85
Sensitivity	0.924	0.582	0.667	0.730
Specificity	0.308	0.583	0.491	0.466
Weighted composite				
AUC (95% CI)	0.714 (0.656, 0.772)	0.567 (0.551, 0.583)	0.638 (0.568, 0.708)	0.568 (0.548, 0.587)
Optimal threshold	− 1.109	0.41	0.22	− 0.30
Sensitivity	0.712	0.589	0.487	0.830
Specificity	0.620	0.518	0.765	0.291
RIAIR				
AUC (95% CI)	0.574 (0.498, 0.650)	0.649 (0.584, 0.713)	0.613 (0.596, 0.631)	0.649 (0.628, 0.670)
Optimal threshold	0.803	1.03	0.790	0.799
Sensitivity	0.379	0.513	0.419	0.429
Specificity	0.793	0.731	0.768	0.788
Combined Weighted composite and RIAIR				
AUC (95% CI)	0.736 (0.667, 0.806)	0.705 (0.640, 0.770)	0.623 (0.606, 0.660)	0.659 (0.637, 0.681)
Optimal threshold	Composite < − 0.1 AND RIAIR < 2.7	Composite < 0.9 OR RIAIR < 0.7	Composite organization < 1.6 OR RIAIR < 0.8	Composite < − 0.5 OR RIAIR < 1.8
Sensitivity	0.676	0.547	0.479	0.653
Specificity	0.662	0.880	0.711	0.575
Combined Unweighted composite and RIAIR				
AUC (95% CI)	0.707 (0.636, 0.777)	0.659 (0.593, 0.726)	0.645 (0.628, 0.662)	0.668 (0.646, 0.690)
Optimal threshold	Composite < 3 AND RIAIR < 2.7	Composite organization < 0.1 OR RIAIR < 0.7	Composite organization < − 3 OR RIAIR < 0.6	Composite < − 2.6 OR RIAIR < 0.7
Sensitivity	0.896	0.538	0.646	0.657
Specificity	0.437	0.722	0.575	0.595

As another important aim of the study, we also compared the predictive power of ROC curves associated with the organization variables with the more established predictor of classic foraging MVT, the RIAIR (the Ratio Instantaneous intake rate / Average Intake Rate; see Methods). According to MVT, optimal foragers would leave a patch as soon as the instantaneous intake rate drops below the average intake rate of that trial. If the instantaneous rate is higher than the average rate, the RIAIR takes values over 1 (staying optimal decision). If the instantaneous rate equals the average rate, the RIAIR takes the value of 1 (optimal moment to leave). If the instantaneous rate is lower than the average rate, the RIAIR is less than 1 (overharvesting).

As we will see in detail in each study and condition, in general, composite organization variables are better predictors than the organization variables alone, although in feature condition from Study 3 the Mean ITD outperforms the composite. We also observe that the composite organization tends to have more sensitivity than the RIAIR, while the RIAIR usually has more specificity than the organization. In this context, this means that the organization measures seem to be better predictors of “leaving decisions”, while the RIAIR seems better at predicting “staying decisions”. Thus, the organization indicators, isolated or in composite variables, outperform the RIAIR to predict the leaving decision (sensitivity), and, in turn, the RIAIR outperforms the organization measures to predict the decision of staying in the patch (specificity). On the other hand, the optimal thresholds show that the best predictions of leaving or staying decisions are slightly larger than 1 in Study 1 (except the fastest condition), as well as the hard memory load condition in Study 2, and roughly at 0.7 in the easiest conditions in studies 2 and 3 (a bit slower probably due to data coming from younger observers of those studies). Optimal MVT would predict that the observers should leave the search when the instantaneous intake rate equals the average intake rate, that is, as soon as the RIAIR equals to 1: In other words, our results show that, in general, observers tend to spend the same or more time than predicted by OFT, overharvesting in important parts of the datasets analyzed here.

Seeing these findings, a relevant question arises: Could we combine the virtues of these two groups of predictors (new organization measures and classic MVT) to obtain a single criterion to accurately predict both “staying” and “leaving” decision in foraging? If so, combining them could help increasing the predictability of the moment to leave a search within a mathematical method using ROC curves, and importantly, potentially implementing future systems to automatically predict the optimal leaving instant in these types of tasks.

We used two approaches to combine the organization variables and the RIAIR in a single ROC curve: a “second-order composite” variable, and “combining ROC curves”. The first approach did not show reliable results (see Methods).

The second approach consists of combining the two ROC curves associated to the weighted composite variable and the RIAIR. We combined them into a single ROC curve, following the method proposed by Haker et al. (2005), also described in the Methods sections. Here, we show the results of the combination of composite and RIAIR curves. Thus, the combining composite and RIAIR unitary ROC curve, compared to the weighted composite and the RIAIR curves, are shown in Figs. 8, 9 and 10, respectively, for studies 1, 2, and 3.

#### Results by conditions for each study

Study 1. Table 2 shows that predictors are in general slightly (but not significantly) better predictors in static than in dynamic conditions. Also, the unweighted composite organization variables are slightly better predictors than the weighted variables. Interestingly, in the slow dynamic condition of study 1, the RIAIR is a good predictor. As we will see later, this result does not replicate in any other condition in study 1 or in any other of the studies here analyzed (remember that in studies 2 and 3 the items were moving at the same speed than in the slow dynamic condition of study 1). Furthermore, combining the composite organization variables with the RIAIR improved the predictive power of the variables; this effect is more pronounced in the slow dynamic condition, perhaps because in this condition the RIAIR is a better predictor than in the other speed conditions.

Study 2. Table 3 shows the values associated with the ROC curves for each individual organization index, the composite organization factor, the RIAIR, and the combination of composite + RIAIR curves, for study 2. Figure 9 shows the corresponding ROC curves. As we can see in Fig. 9 and Table 3, the low memory load (easier) condition yielded more predictive criterion in both age groups, and they were slightly more sensitive for adolescents and adults than for children. The unweighted composite was again a better predictor than the weighted composite. Combined ROC curves had similar predictiveness to the unweighted composite in the groups with memory load 2, slightly improving the sensitivity and specificity of the predictions in the memory load 7 group. Again, the combined curve equals the organization composite or RIAIR or significantly outperform both (see Table 3, memory load 7 for the older observer group), suggesting the combined curve as the best predictor of quitting rules also in this study when considering different conditions at different ages.

Study 3. The first remarkable result derived from the analyses of study 3 (Table 4 and Fig. 10) is that there are no essential differences for age under conjunction, more difficult conditions, while there is a clearer effect of age for feature condition. Also, in a less clear manner than in the two former studies, in this study the unweighted composite only outperformed the weighted composite in the conjunction condition. For feature conditions, the weighted composite organization variable for children below 10 years old seems a better predictor than the RIAIR, just like the general results showed for the whole sample. In fact, for children under 10 years old in feature conditions, the obtained thresholds for the weighted composite organization variable predict quite well leaving decisions, despite having high values. Interestingly, this trend reverses for adolescents and young adults. Regarding conjunction conditions, the two ROC curves for weighted composite organization and RIAIR are very similar. The ROC curve combining the composite organization and the RIAIR shows slightly better AUC values and their combined thresholds have slightly more sensitivity than the weighted composite (for feature in children) and more specificity than the RIAIR (for feature in adolescents and young adults). Importantly, the combined composite-RIAIR ROC curve is the best predicting quitting behavior in all conditions, including feature conditions for older observers and conjunction ones for children, in which it clearly outperforms the composite in both cases (see confidence intervals in Table 4). That is, although the combined curve seemed to be similar to the organization composite in the general analysis, when considering different factors, it seems to work significantly better at some of those conditions, being always the best predictor.

## Discussion

We had two main goals in the present work: first, studying whether classic organization measures in search (best-r, Mean ITD, PAO, and the intersection rate) can be good predictors determining the moment to abandon a search/foraging, and comparing their predictiveness with classic foraging intake rates. If organization measures are good at predicting quitting rules, a second important goal was to determine whether a single organization-composite variable could predict good enough those stopping rules in search. Our aim was to obtain a single predictive factor by combining previous organization indexes. The results show clear evidence for the organization measures as good predictors of quitting rules in different types of foraging and under different conditions. Importantly, we show that a single organization factor can outperform classic intake rates predicting the best moment to leave a search in humans. Moreover, joining classic MVT indexes with organization factors seems to be the best way to explain quitting rules in search, especially when considering diverse conditions and factors within the studies.

The parallel analysis and PCA results in the three studies support a unitary organization factor. Indeed, Woods and Mark (2007) already reported convergent validity of those organization indexes, although with subjective-reported measures, but in the same line as our findings. Still, the factors loading for best-r and Mean ITD were lower than PAO and intersection rates. Indeed, they are all conceived to capture diverse aspects of organization: best-r captures scanner-like patterns (up-down, left–right), mean ITD and PAO are designed to capture the “shortest-optimal” paths, while intersection rate captures patterns avoiding revisitations. Our ROC curves showed that Mean ITD and best-r have a limited predictive power for quitting behavior (those factors that had lower loading levels in the PCA), while PAO and intersection rates are more discriminative (having higher loads in the PCA). Importantly, the composite organization variable considers those loads derived from the PCA, showing it as an adequate predictor of leaving decisions according to ROC curves results. Although PAO and intersection rates show similar predictive values (still slightly worse than composite factors), since our composite variable takes into account all organization indexes and their diverse aspects in organization in search, we suggest the composite as the best way to predict quitting rules in our tasks. While more research is needed in a more variety of foraging and search tasks, the results of these three studies with almost 400 observers under different conditions and ages show a clear good potential of organization to predict quitting rules in search, and particularly, the use of a composite variable comprising diverse aspects of organization at once.

Another critical result in our study is that the composite factor, particularly the unweighted composite, clearly outperforms classic MVT intake rate at predicting when to quit the search. MVT proposed that foragers tend to optimize intake rates, suggesting that the optimal moment quitting is that at which the instantaneous intake rate meets the average intake rate (RIAIR index). According to MVT, the RIAIR would be a good predictor if lying around a threshold of 1. The most discriminative threshold obtained here lies around a ratio of 0.7, suggesting that in general observers tend to overharvest. This result is consistent with similar overharvesting results shown in the literature in the last decade with humans (Gil-Gómez de Liaño et al., 2022; Harhen & Bornstein, 2023; Wiegand et al., 2019; Wolfe, 2013). In fact, Harhen and Bornstein (2023) suggest that this “human-overharvesting” may be adaptive to *learn* about patch profitability for future searches, which could indeed explain why our composite organization variable seems a better predictor for quitting rules. Indeed, developmental differences in quitting rules have shown that as we get older (and probably, we *learn* more about our environment) we tend to overharvesting, at least in most profitable environments (Lloyd et al., 2023). However, in study 1, with only adult participants, the most predictive RIAIR threshold was placed around 1, and even above 1, except in the fastest dynamic condition. With that in mind, search organization could play a key role in the *search learning* process, explaining why it would be a better predictor of quitting rules in humans. Actually, age and conditions comparisons in studies 2 and 3 (see Figs. 9 and 10, and Tables 3 and 4) based on the organization indicators show some differences with adolescents and young adults exploiting patches more than children. This over exploiting trend for young adults is in agreement with the *cooling-down* effect described by Lloyd et al. (2023): As observers get older, they tend to learn from experience with more exploiting behaviors in foraging. Nonetheless, in the samples with only adults in study 1, this was not always the case, so more research is needed to test the cooling-down effect hypothesis.

Interestingly, the RIAIR has also shown larger specificity than organization measures: It is better at predicting “staying rules” rather than “quitting rules”. Complementarily, the composite organization factor has larger sensitivity, being better at predicting “quitting” than “staying” rules. Thus, we explored a combination index calculating a new ROC curve associated with RIAIR and the weighted organization factor. The combined ROC outcome had slightly improved the predictiveness compared with the two originally proposed predictors in the general results, but significantly better for certain conditions in the studies, being always at least as good as the composite organization, and for several conditions, significantly better.

Finally, conjunction foraging tested in study 3 is way more difficult than feature foraging, and therefore, leads to poorer organization indexes (Bella-Fernández et al., 2024; Kristjánsson et al., 2020; Ólafsdóttir et al., 2021), and poorer predictive power. Similarly, high memory loads from study 3 make the task more difficult, and the predictability of the quitting criterion is also poorer than with lower memory loads. Still, the results of organization indexes show better predictability than MVT intake ratios, showing that following a threshold approach based on organization could be more functional to detect age differences in quitting rules, as shown in studies 2–3. Actually, the combination of organization composite and classic MVT RIAIR seems to be the best way to predict quitting rules under all conditions tested here from study 1 through 3.

Regarding the organization measures, we have already mentioned that the four indicators used in this article are widely used and showed evidence of validity through subjective measures. However, they have been mostly used in static contexts, while we only recently applied them in dynamic contexts. Previous results (Bella-Fernandez et al., 2024) have shown the robustness and reliability of the indexes in dynamic foraging. The consistency of the present results together with the comparisons between static and dynamic foraging, strengthen the capability of the indexes measuring organization in both dynamic and static foraging. Another question may arise about *how* these indexes might work in dynamic foraging and *what* they can tell us to understand search in these types of tasks. Do participants anticipate target movements in this changeable-dynamic environment or they just pick the nearest target at any time after they have found a previous one? With the current data, it is not possible to determine whether the participants anticipate target movements or pick the nearest target in dynamic foraging. What we know is that there is organization at the beginning of each trial that becomes more disorganized at the end (see also Bella-Fernández et al., 2024), before moving to a new one. However, we could speculate that participants might be doing a mixture of both anticipate-and-picking the closest target: there is evidence elsewhere (Kosovicheva et al., 2020) of anticipation of 1–2 targets in advance when picking the current target, although in dynamic contexts this anticipation seems to be more limited (Blanco et al., 2009). More research is needed to determine how this organization works, particularly in motion foraging, but the present study show evidence that even in motion foraging, the study of organization seems relevant to the field.

Definitely, more research is needed to test how organization, intake rates, and search learning processing can affect quitting rules in search in a wider variety of search task, but all results found in the present paper critically demonstrate that all of them have potential to jointly predict leaving decisions in search (among other things mentioned right before). Other studies like those based on Bayesian models (e.g., Clarke et al., 2022, or Le et al., 2023) might have potential to understand organization in foraging. Although they are quite new and have based the research in understanding how the search is done, they could definitely be applied in the study of organization in foraging. Certainly, our findings open a new research line for studying the decision of staying or leaving a visual search, based on the organization and/or a combined measure of organization and intake rates based on MVT to better predict when the observers leave a search that could also join these new Bayesian models and others.

## Supplementary Information

Additional file 1.

## Data Availability

The data that support the findings of this study are not openly available due to reasons of sensitivity and are available from the corresponding author upon reasonable request.

## Footnotes

1 Serious video games are video games designed with other purposes than entertainment, such as cognitive assessment, therapy or research (e.g. Prpic et al., 2019).

2 The Mean ITD can be calculated from the second target; with two targets, the Mean ITD would simply be the distance between those two targets. The best-r can also be calculated from the second target, but with two targets it would always be 1, and it would only vary from the third target. PAO can also be calculated from the second target, but it would be 0 with less than three targets. Regarding the intersection rate, it would be 0 with less than four targets. However, calculating the indexes under those conditions did not significantly change any conclusion, so we decided to make calculations identical for all indexes from the third target.

3 Another possibility was performing a Confirmatory Factor Analysis with a unidimensional factor structure, but the assumptions of data normality and independence between observations were not met in our case.
