# Supplementary material for: One factor to bind them all: visual foraging organization to predict patch leaving behavior with ROC curves
Source: Cogn Res Princ Implic. 2025 Apr 5;10:16. doi: 10.1186/s41235-025-00624-7 (PMC11972240; doi:10.1186/s41235-025-00624-7)
Supplement: Supplementary file 1 — Additional file 1. [file 41235_2025_624_MOESM1_ESM.docx]

Combining ROC corves through second-order composite variable: a failed approach

This approach consists of combining the composite organization variable obtained from the weighted sum of the organization indicators with the RIAIR. The method is the same as the one described above and summarized in Equation 2. The result would be a “second-order composite” variable. We tried this approach on data from Study 3, for no particular reason.

We performed a parallel analysis with the four organization variables and the RIAIR in order to assess the plausibility of this variable or, in other words, the unidimensionality of this construct. The result of the parallel analysis suggested that the combination of the composite organization variable and the RIAIR can be considered unidimensional, indicating one dimension to retain (Adjusted Eigenvalue: 2.59).

We then performed two different PCAs: a PCA with the four organization variables and the RIAIR (a total of 5 variables), and another PCA with the composite organization variable and the RIAIR (a total of 2 variables). The proportion of variance explained on each PCA was 45.6% and 57.9%, respectively. The loadings for the PCA in five variables were 0.248 (best-r), -0.230 (Mean ITD), -0.471 (PAO), -0.571 (Intersection rate), and 0.582 (RIAIR), while the loadings for the PCA with two variables were 0.264 (composite variable) and 0.964 (RIAIR).

Their respective ROC curves have AUCs of 0.677 and 0.622. Thus, we consider that this approach is not fruitful to obtain decision criteria which combines the best properties of organization and RIAIR when predicting the decision of staying or leaving, because they simply do not improve the predictability power of quitting rules; at least not following this approach for combining organization and MVT indexes.

Organization measures through time

We tested the behavior of organization measures through time and relative to the target prevalence. To this end, we fitted linear mixed models with each organization measure as dependent variable, the participant ID and the trial number as random effects, and the target prevalence or the number of targets already picked as fixed effect factors; these fixed effect factors were tested in separate models. Table 1 shows the results of the model fitting. In general, we can see a robust effect of depletion on organization: as target prevalence become lower (it is, as targets become scarcer), the organization decreases. We can also see an effect of intra-trial time: as a trial goes on, the organization decreases (except for the Mean ITD). These models alone do not allow us to establish causal relationships, but they allow to obtain evidence of the general decrease in organization.

Table S1: Linear mixed models. The random variables were the participant ID and the trial number. All values are t statistics.

|  | Fixed effect | | | |
| --- | --- | --- | --- | --- |
|  | Target prevalence | | Number of targets picked up | |
| Organization variable (dependent variable) | Estimation | t statistic | Estimation | t statistic |
| Best-r | 1.89 | 54.5 | -0.017 | -59.43 |
| Mean ITD | -523.96 | 27.84 | 0.217 | 1.262 |
| PAO | -241.580 | -43.31 | 2.45 | 52.37 |
| Intersection rate | -1.81 | -56.24 | 0.019 | 74.43 |

Confidence interval for combined ROC curves

Hanley and McNeil (1982) described a method to estimate 95% confidence intervals for ROC curves. We applied this methos to estimate the confidence intervals of the combined ROC curves because the method applied to obtain them does not include any of such estimations.

Equation S1 describes the confidence interval as a function of the standard error (se) and the significance. In all of these equations, AUC is the AUC estimated for a certain ROC curve. The significance and its related z value are straightforward, but the calculation of the se is not. Equation S2 described the calculation of the se. In this context, n_1_ is the number of data associated with a decision of “leaving” and n_2_ is the number of data associated with a decision of “staying”. Equation S2 contains three parameters, q_0_, q_1_, and q_2_, whose calculation is in turn described in Equations S3 to S5, respectively.

$CI\left( AUC \right)=AUC\pm se\cdot z_{1-\frac{\alpha}{2}}$ [S1]

$se=\sqrt{\frac{q_{0}+\left( n_{1}-1 \right)\cdot q_{1}+\left( n_{2}-1 \right)\cdot q_{2}}{n_{1}\cdot n_{2}}}$ [S2]

$q_{0}=AUC\left( 1-AUC \right)$ [S3]

$q_{1}=\frac{AUC}{2-AUC}$ [S4]

$q_{2}=\frac{2\cdot{AUC}^{2}}{1+AUC}$ [S5]
